# Quinine in Coffee

**Published:** 1847-08

**Authors:** 


7. *Quinine in Coffee.*—A Parisian medical student, M. des Veuves, has been recommending the administration of quinine in an infusion of coffee, as the best means of concealing its bitterness, and as not impairing its medicinal powers. There is a warm discussion as to whether this is a discovery. It appears, however, not to be new but that the young man has the honor of having directed the attention of the medical faculty in Paris to this useful mode of giving quinine, which had been almost entirely overlooked. It is worthy of a trial in this country.—*Lond. Lancet* in *Bost. Med. and Surg. Jour.*

